# Perception of parents and caregivers regarding the impact of malocclusion on adolescents’ quality of life: a cross-sectional study

**DOI:** 10.1590/2177-6709.21.6.074-081.oar

**Published:** 2016

**Authors:** Lucas Guimarães Abreu, Camilo Aquino Melgaço, Mauro Henrique Abreu, Elizabeth Maria Bastos Lages, Saul Martins Paiva

**Affiliations:** 1 PhD student, Universidade Federal de Minas Gerais (UFMG), Department of Pediatric Dentistry and Orthodontics, Belo Horizonte, Minas Gerais, Brazil.; 2 Postdoctorate fellow, Universidade Federal de Minas Gerais (UFMG), Department of Pediatric Dentistry and Orthodontics, Belo Horizonte, Minas Gerais, Brazil.; 3 Professor, Universidade Federal de Minas Gerais (UFMG), Department of Community and Preventive Dentistry, Belo Horizonte, Minas Gerais, Brazil.; 4 Professor, Universidade Federal de Minas Gerais (UFMG), Department of Pediatric Dentistry and Orthodontics, Belo Horizonte, Minas Gerais, Brazil.

**Keywords:** Parents, Caregivers, Malocclusion, Adolescent, Quality of life

## Abstract

**Objective:**

The objective of this article was to assess the perception of parents and caregivers regarding the impact of malocclusion on adolescents’ oral health -related quality of life (OHRQoL).

**Methods:**

This cross-sectional study consisted of a sample of 280 parents/caregivers of 11 and 12-year-old adolescents who answered the Parental-Caregiver Perceptions Questionnaire (P-CPQ). Parent-assessed quality of life of adolescents was the dependent variable. The main independent variable was adolescents’ malocclusion which was diagnosed by means of the Dental Aesthetic Index (DAI). Based on DAI cut-off points, adolescents were classified into four grades of malocclusion, with different orthodontic treatment recommendations assigned to each grade: no need/slight treatment need, elective treatment, highly desirable treatment and mandatory treatment. Adolescents’ age and sex, as well as family monthly income, were considered as confounding variables. Statistical analysis involved descriptive statistics, bivariate analyses, and Poisson regression with robust variance.

**Results:**

Of the 280 parents/caregivers initially accepted in this study, 18 refused to answer the P-CPQ. Therefore, 262 individuals participated in this assessment, providing a response rate of 93.5%. The severity of adolescents’ malocclusion was significantly associated with a higher negative impact on parents’/caregivers’ perception on the oral symptoms (*p*< 0.05), functional limitations (*p* < 0.001), emotional well-being (*p* < 0.001), and social well-being (*p* < 0.001) subscale scores as well as on the overall P-CPQ score (*p* < 0.001), even after having been adjusted for the controlling variables.

**Conclusions:**

Parents/caregivers reported a negative impact of malocclusion on adolescents’ OHRQoL. Increased severity of malocclusion is associated with higher adverse impact on OHRQoL.

## RESUMO

**Objetivo:**

o objetivo deste artigo foi avaliar a percepção de pais/cuidadores com relação ao impacto da má oclusão na qualidade de vida relacionada à saúde bucal (QVRSB) de adolescentes.

**Métodos:**

este estudo transversal consistiu de uma amostra de 280 pais/cuidadores de adolescentes com 11 a 12 anos que responderam o *Parental-Caregiver Perceptions Questionnaire* (P-CPQ). A percepção de pais/cuidadores com relação à qualidade de vida dos adolescentes foi considerada a variável dependente. A variável independente principal foi a má oclusão dos adolescentes, que foi diagnosticada por meio do Índice Estético Dental (IED). Com base nos pontos de corte do IED, os adolescentes foram classificados em quatro categorias de má oclusão, com diferentes recomendações de tratamento ortodôntico atribuídas a cada uma dessas categorias: sem necessidade ou com leve necessidade de tratamento; tratamento eletivo; tratamento altamente desejável; e tratamento obrigatório. A idade e o sexo dos adolescentes e a renda familiar mensal foram consideradas variáveis de confusão. A análise estatística envolveu estatística descritiva, análise bivariada e regressão de Poisson com variância robusta.

**Resultados:**

dos 280 pais/cuidadores inicialmente aceitos nesse estudo, 18 se recusaram a responder ao P-CPQ. Portanto, 262 indivíduos participaram dessa avaliação, resultando em uma taxa de resposta de 93,5%. A gravidade da má oclusão dos adolescentes foi significativamente associada com um impacto mais negativo na percepção de pais / cuidadores nas subescalas sintomas bucais (*p* < 0,05), limitações funcionais (*p* < 0,001), bem-estar emocional (*p* < 0,001) e bem-estar social (*p*< 0,001), mesmo após o ajuste para as variáveis de confusão.

**Conclusões:**

os pais/cuidadores relataram um impacto negativo da má oclusão na QVRSB dos adolescentes. Quanto mais grave a má oclusão, mais adverso é o impacto dessa condição na QVRSB.

## INTRODUCTION

Oral health-related quality of life (OHRQoL) has been defined as the extent to which oral outcomes affect individuals’ oral functioning, psychological well-being, and social well-being.^1^ In recent decades, patient-centered tools focusing on individuals’ self-perception have been used to assess the impact of oral conditions on their quality of life.^2^ Traditional methods to evaluate oral health based on clinical standards are undeniably important. However, they have proven to be limited, since they do not consider the psychosocial aspects of health and should, therefore, be supplemented by subjective measures.^3^ More recently, efforts have been made to develop measures of OHRQoL that would be suitable for use on children and adolescents. The introduction of OHRQoL has unveiled a new perspective by suggesting how oral outcomes impact the lives of young patients and their families in general.^4^

The results of a systematic review showed that malocclusion negatively impacts adolescents’ OHRQoL.^5^ In general, increased severity of the condition is associated with a higher impact on the individuals’ quality of life.^6^ The primary effect of malocclusion on adolescents’ OHRQoL has most commonly been recognized in the domains of emotional and social well-being.^5^ Adolescents clearly attribute high importance to an attractive dental appearance, and irregularities in the position of the teeth may reduce social acceptance^7^ and induce low self-esteem,^8^ which can ultimately deteriorate quality of life through psychosocial pathways. Moreover, evidence shows that malocclusion can compromise adolescents’ chewing and speech capabilities.^9^

Despite being well-documented from the adolescents’ perspective, the impact of malocclusion on those individuals’ OHRQoL using the views of their parents/caregivers has, though, received little scientific attention to date.^10^ Factors influencing parental attitude and behavior related to adolescents’ oral outcomes warrant a broader and more in-depth investigation.^11^ For many reasons, health care providers should consider caregivers’ beliefs and values regarding symptoms, oral function, and well-being when guiding the families of adolescents with malocclusion. First, information provided by parents/caregivers can serve to complement existing reports provided by adolescents.^10^ Second, parents/caregivers may be aware of some key orthodontic variables regarding their sons/daughters, and these attributes may have an impact on both informed consent and satisfaction with the orthodontic treatment provided.^12^ Finally, data collected from parents/caregivers are also relevant because these individuals are often the main decision-makers regarding adolescents’ health, and their perceptions exert major influence on treatment choices.^13^

Therefore, the aim of this study was to evaluate parents’/caregivers’ views regarding the impact of malocclusion on the OHRQoL of Brazilian adolescents, by means of the Parental-Caregiver Perceptions Questionnaire (P-CPQ).^14^ It was hypothesized that malocclusion is not associated with impairment of adolescents’ OHRQoL when the perceptions of parents/caregivers are assessed.

## METHODS

This research was a cross-sectional study intended to assess parents’/caregivers’ perception regarding the impact of malocclusion on Brazilian adolescents’ OHRQoL. This article followed the Strengthening the Reporting of Observational Studies in Epidemiology (STROBE) guidelines.^15^

## PARTICIPANTS, SETTING, PERIOD OF RECRUITMENT AND ELIGIBILITY CRITERIA

A consecutive sample of parents/caregivers of 11 and 12-year-old adolescents was selected. In this study, participants were identified through the dental screening program of the Division of Orthodontics at Universidade Federal de Minas Gerais (UFMG) in September 2013. This program consists of oral examination of adolescents who were referred to the School of Dentistry to find out whether or not they needed orthodontic treatment. Adolescents, along with their parents/caregivers, were invited to participate. For inclusion in the sample, parents/caregivers needed to be fluent in Portuguese. The exclusion criteria consisted of parents/caregivers of adolescents with dental caries, history of dental trauma, poor gingival health, craniofacial anomalies, and cognitive disorders, as well as those who had undergone any dental treatment within the past three months. Calibration for dental caries diagnosis was performed according to World Health Organization (WHO) criteria.^16^ The Andreasen et al^17^ classification was used for traumatic dental injury, whereas the criteria developed by Loe^18^ were used to analyze gingival diseases.

## SAMPLE SIZE CALCULATION

Based on a pilot study, sample size was calculated to establish a power of 80% and a confidence interval of 95%. The following parameters were also considered: standard deviation of the mean overall P-CPQ score in the unexposed group (parents/caregivers of adolescents with no orthodontic treatment needs) of 11.7, and a standard deviation of the mean overall P-CPQ score in the exposed group (parents/caregivers of adolescents with orthodontic treatment needs) of 16.7. The difference to be detected was set at 4.3 as a mean P-CPQ score difference between groups. Minimum sample size to satisfy the requirements was estimated to be of 237 individuals. Taking into consideration non-response/attrition, the final sample size was 280 parents/caregivers of adolescents.

## ETHICAL CLEARANCE

All aspects of this study, including methods to obtain informed consent and agreement from participants (parents/caregivers and adolescents), were independently reviewed and deemed to be ethical by the Research Ethics Board of Universidade Federal de Minas Gerais (UFMG) (Protocol #0421.0.203.000-11). This study was conducted in accordance with the principles for medical research involving human subjects set forth in the Helsinki Declaration. Collected data remained anonymous and confidential. 

## MEASURES

The outcome variable was defined as the parents’ / caregivers’ perception of the impact of malocclusion on adolescents’ quality of life. Adolescents’ malocclusion was the main independent variable. Family monthly income, as well as adolescents’ age and sex, were defined as confounding variables.

## OHRQOL TOOL

Quality of life data were collected through the Parental-Caregiver Perceptions Questionnaire (P-CPQ)^14^ which was developed in Canada, translated, and verified for use in the Portuguese language.^19^ It consists of 31 questions distributed into four subscales: oral symptoms (OS), functional limitations (FL), emotional well-being (EW), and social well-being (SW). Each question has five response options: “never” = 0; “once or twice” = 1; “sometimes” = 2; “often” = 3; and “every day or almost every day” = 4.^16^ A “don’t know” option is also provided. The method used to manage this option was to calculate, for each participant, mean scores based on items with responses other than “don’t know.” Thus, the scores were adjusted for the number of items that contributed to the score.^11^ The overall score is computed by adding up all questions’ scores and ranges from 0 to 124. Scores for each of the four subscales can also be computed separately. A higher score denotes a greater negative perception on the part of parents/caregivers as regards their adolescents’ OHRQoL.^14^ ^,^ ^19^ The P-CPQ shows reliability and validity. The former reflects the degree to which a test score is free from measurement errors. The latter refers to the appropriateness, significance and usefulness of specific inferences drawn from test scores, which is, therefore, considered a process of accumulating evidence based on such inferences.^20^ Parents/caregivers self-completed the questionnaire separately in order to ensure that adolescents did not influence their answers in any way. The information was provided in a quiet area of the university clinic with a researcher available to clarify any questions. The questions address the frequency of events regarding problems with adolescents’ teeth, lips, jaws, or mouth, considering a self-reported recall of the previous three months. For this reason, administration of the questionnaires was limited to parents/caregivers of adolescents with no dental disease other than malocclusion and no dental treatment in a period of time shorter than this interval, thereby avoiding any bias that could have occurred if the three-month time frame had not been considered.

## MALOCCLUSION ASSESSMENT

Adolescents were clinically examined to assess malocclusion and to determine their orthodontic treatment needs by means of the Dental Aesthetic Index (DAI). This cross-cultural index consists of ten occlusal characteristics related to dentofacial anomalies according to three components: dentition (number of missing incisors, canines, and premolars); crowding and/or spacing (crowding in the incisal segments, spacing in the incisal segments, midline diastema, largest anterior irregularity on the maxilla, and largest anterior irregularity on the mandible); and occlusion (maxillary overjet, mandibular overjet, anterior open bite and anterior posterior molar relationship). The scores for each occlusal characteristic are multiplied by their specific weight and a constant value of 13 is added to obtain the final DAI score for each participant. Based on DAI cut-off points, adolescents were classified into four grades of malocclusion with distinct orthodontic treatment recommendations assigned to each grade: normal or minor malocclusion / no need or slight treatment needed (DAI ≤ 25), definite malocclusion/elective treatment (26 ≤ DAI ≤ 30), severe malocclusion/highly desirable treatment (31 ≤ DAI ≤ 35), and very severe malocclusion/mandatory treatment (DAI ≥ 36).^21^

Calibration exercise was carried out before beginning the study, to ensure reliable data collection. Two dentists were calibrated for the use of DAI. The calibration process consisted of both theoretical and clinical training. The theoretical step involved a discussion on the criteria used to diagnose malocclusion. The clinical step involved the examination of 15 adolescents who were not included in the main study. Examinations were performed by each of the two dentists separately to calculate interexaminer agreement. Ten days later, adolescents were reassessed to calculate intraexaminer agreement. Kappa values ranged from 0.84 to 0.90 for both inter- and intraexaminer agreement. As Kappa coefficients were very good, examiners were considered apt to conduct this epidemiological study.

## FAMILY MONTHLY INCOME

Household income was categorized in terms of the Brazilian Monthly Minimum Wage (BMMW) which corresponded to US$ 325.00 at the time of the study and was established as the monthly income of all economically active members of the family. For statistical analysis, household income was categorized as follows: parents/caregivers of adolescents whose families have a monthly income equal to or lower than 1 BMMW, > 1 to ≤ 3 BMMWs, > 3 to ≤ 5 BMMWs or higher than 5 BMMWs.

## PILOT STUDY

Following the calibration process, a pilot study, conducted with adolescents and their parents/caregivers who did not participate in the main study, was carried out in order to calculate sample size as well as to test the administration of questionnaires and dental examination of adolescents. The results of the pilot study showed that changes in the proposed data collection protocol were unnecessary.

## STATISTICAL ANALYSIS

All statistical analyses were performed by means of the Statistical Package for the Social Sciences (SPSS for Windows, Version 22.0, SPSS Inc., Chicago, IL, USA). Missing data were handled using mean imputation. Descriptive statistics were calculated, followed by the application of a nonparametric bivariate analysis. Mann-Whitney and Kruskal-Wallis tests were used to compare the overall and subscale P-CPQ scores for malocclusion, family monthly income, and adolescents’ age and sex. Poisson regression with robust variance was used to perform multivariate analysis. Overall and subscale P-CPQ scores were compared in terms of the robust rate ratio and the respective 95% confidence intervals for the malocclusion categories. Malocclusion was incorporated into the model and adjusted for confounding variables (family monthly income as well as adolescents’ age and sex). The confounding variables were incorporated into the model based on statistical significance (*p*< 0.20) and epidemiological relevance. The statistical significance level for the final model was set at 5% (*p*< 0.05). 

## RESULTS

**Figure 1:**
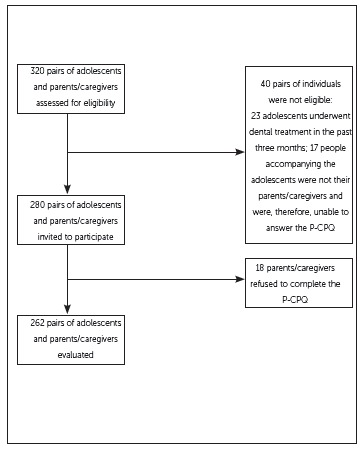
Flow diagram of the study.

A total of 262 pairs of adolescents and their parents / caregivers participated in the present study, corresponding to 93.5% of the total selected pairs based on sample size calculation. Reasons for non-response were that 18 parents/caregivers refused to participate. The number of missing and “don’t know” responses was lower than 1%. The flow diagram of the study is depicted in Figure 1. Mean age of adolescents was 11.7 years. Most respondents were adolescents’ mothers. Socio-demographic data and adolescents’ orthodontic treatment needs are presented in Table 1.

**Table 1:** Socio-demographic characteristics of the sample and adolescents’ orthodontic need.

	n (%)
Adolescents’ sex	
Male	120 (45.8)
Female	142 (54.2)
Adolescents’ age (years)	
11	96 (36.6)
12	166 (63.4)
Respondents	
Mother	205 (78.2)
Father	46 (17.6)
Other	11 (4.2)
Family monthly income (BMMW)	
≤ 1 BMMW	20 (7.6)
> 1 to ≤ 3 BMMWs	129 (49.3)
> 3 to ≤ 5 BMMWs	93 (35.5)
> 5 BMMWs	20 (7.6)
Adolescents’ malocclusion (DAI)	
≤ 25	98 (37.4)
26 to 30	98 (37.4)
31 to 35	47 (17.9)
≥ 36	19 (7.3)

BMMW = Brazilian Monthly Minimum Wage.

DAI = Dental Aesthetic Index

Table 2 displays the mean (SD), minimum and maximum values of P-CPQ obtained for the entire sample. Table 3 shows the mean (SD) overall and subscale P-CPQ scores according to adolescents’ malocclusion and confounding variables. The overall and subscale P-CPQ scores varied when the different categories of adolescents’ malocclusion were compared. The severity of malocclusion was significantly associated with higher mean P-CPQ scores in the OS (*p* = 0.003), FL (*p*= 0.021), EW (*p* = 0.007), and SW (*p* = 0.002) subscales as well as in the overall score (*p* = 0.003).

**Table 2:** Mean (SD), minimum and maximum values of P-CPQ obtained considering the entire sample

	P-CPQ range	Mean (SD)	Minimum	Maximum
OS	0 - 24	4.79 (2.63)	0	16
FL	0 - 32	4.85 (3.61)	0	21
EW	0 - 28	5.18 (3.89)	0	23
SW	0 - 40	5.41 (5.46)	0	30
OL	0 - 124	20.19 (12.30)	2	80

OS = oral symptoms; FL = functional limitations; EW = emotional well-being; SW = social well-being; OL = overall score; SD = standard deviation.

**Table 3:** Mean (SD) P-CPQ overall and subscale scores according to independent variables.

	OS mean (SD)	FL mean (SD)	EW mean (SD)	SW mean (SD)	OL mean (SD)
Adolescents’ sex					
Male	5.03 (2.72)	5.09 (3.51)	5.10 (4.25)	5.10 (5.41)	20.33 (12.42)
Female	4.58 (2.55)	4.65 (3.69)	5.24 (3.57)	5.68 (5.51)	20.08 (12.25)
*p*-value*	0.125	0.200	0.319	0.520	0.807
Adolescents’ age (years)					
11	4.66 (2.80)	5.20 (3.72)	5.78 (4.68)	6.52 (6.68)	22.16 (14.87)
12	4.86 (2.54)	4.66 (3.54)	4.83 (3.32)	4.77 (4.51)	19.05 (10.42)
*p*-value*	0.375	0.200	0.272	0.133	0.292
Family Income (BMMW)					
≤ 1 BMMW	4.80 (3.45)	6.20 (5.37)	7.10 (5.34)	8.10 (7.26)	26.20 (18.47)
> 1 to ≤ 3 BMMWs	4.78 (2.39)	5.29 (3.87)	5.38 (4.14)	5.60 (5.55)	21.05 (12.43)
> 3 to ≤ 5 BMMWs	4.95 (2.74)	4.24 (2.76)	4.71 (3.20)	4.90 (4.87)	18.69 (10.31)
> 5 BMMWs	4.10 (2.82)	3.55 (2.37)	4.10 (2.86)	3.90 (4.83)	15.65 (10.33)
*p*-value**	0.538	0.144	0.262	0.153	0.076
Adolescents’ malocclusion (DAI)					
≤ 25	4.72 (2.54)	4.30 (3.08)	4.41 (3.09)	4.43 (4.40)	17.76 (9.25)
26 to 30	4.69 (2.75)	4.47 (3.32)	4.87 (3.71)	4.76 (4.92)	18.79 (12.17)
31 to 35	5.68 (2.39)	6.40 (4.76)	6.55 (4.79)	7.43 (6.99)	26.06 (15.59)
≥ 36	3.37 (2.45)	5.89 (3.23)	7.32 (4.66)	8.89 (6.48)	25.47 (12.31)
*p*-value**	0.003	0.021	0.007	0.002	0.003

OS = oral symptoms; FL = functional limitations; EW = emotional well-being; SW = social well-being; OL =overall score.

SD = standard deviation.

BMMW = Brazilian Monthly Minimum Wage.

DAI = Dental Aesthetic Index.

*Mann-Whitney test.

**Kruskal-Wallis test.

Table 4 presents the results of multivariate Poisson regression analysis with robust variance. Adolescents’ age and sex as well as family monthly income were incorporated into the model as potential confounding variables. For the overall P-CPQ score, the final model showed the following result: the greater the severity of adolescents’ malocclusion, the higher the negative impact on the perception of parents/caregivers regarding their sons’/daughters’ quality of life (*p* < 0.001). Moreover, in general, parents/caregivers of adolescents with a more severe malocclusion were more likely to have a negative perception of the impact of malocclusion on adolescents’ OHRQoL regarding OS (*p* < 0.05), FL (*p*< 0.001), EW (*p* < 0.001), and SW (*p* < 0.001) subscales. 

**Table 4:** Multivariate Poisson regression model for the association between P-CPQ overall and subscale scores and adolescents’ malocclusion.

	OS Robust RR (95% CI)	FL Robust RR (95% CI)	EW Robust RR (95% CI)	SW Robust RR (95% CI)	OL Robust RR (95% CI)
Malocclusion (DAI)					
≤ 25	1.00	1.00	1.00	1.00	1.00
26 to 30	0.98 (0.86 - 1.11)	1.04 (0.91 - 1.19)	1.12 (0.98 - 1.28)	1.10 (0.97 - 1.26)	1.07 (1.00 - 1.14)*
31 to 35	1.18 (1.01 - 1.37)*	1.45 (1.25 - 1.68)**	1.48 (1.28 - 1.72)**	1.70 (1.47 - 1.95)**	1.45 (1.35 - 1.57)**
≥ 36	0.72 (0.55 - 0.94)*	1.31 (1.06 - 1.63)*	1.60 (1.31 - 1.95)**	1.81 (1.50 - 2.17)**	1.37 (1.24 - 1.52)**

OS = oral symptoms; FL = functional limitations; EW = emotional well-being; SW = social well-being; OL = overall score.

RR = rate ratio.

CI = confidence interval.

DAI = Dental Aesthetic Index.

*p < 0.05, **p < 0.001.

Model adjusted for control variables (sex, age, and family income).

## DISCUSSION

The present study assessed parents’/caregivers’ perception of the impact of malocclusion of adolescents on their OHRQoL. Parents/caregivers reported a negative impact of malocclusion on the overall quality of life of their adolescents. Results were also statistically significant in OS, FL, EW, and SW subscales. To the best of our knowledge, this is the first study that involved parents / caregivers of 11 and 12-year-old adolescents and that used a validated quality of life tool to reach this specific outcome. Similar results were found in previous reports; however, the primary aim of those reports was to validate the P-CPQ in different languages and cultures, using convenience samples and assessing other types of oral conditions, such as dental caries, fluorosis, and gingivitis.^14^ ^,^ ^22^ Therefore, the present study represents a significant contribution to scientific knowledge by unveiling such evidence in a sample of Brazilian adolescents and their respective guardians.

Results from the present study run in direct contrast with those from prior reports assessing the impact of malocclusion on the quality of life of preschoolers.^23^ ^,^ ^24^ In those reports, parents’/caregivers’ views did not indicate any significant impact on children’s OHRQoL. This lack of impact is most likely to the fact that, at this age, children do not prioritize aesthetics, which is a major concern for adolescent groups, especially regarding its impact on the EW and SW subscales.^24^ In addition, more severe cases of malocclusion, such as increased overjet and diastema, which can exert a negative impact on the FL subscale, are more prevalent in mixed and permanent dentitions.^9^ The results of this article could also be compared to the results of a study evaluating orthodontic treatment. A recent assessment showed that parents/caregivers reported an improvement on adolescents’ OHRQoL during fixed appliance therapy with positive effects regarding EW and SW subscales.^25^

The percentage of malocclusion scores, in the present report, was slightly different from another evaluation, which also targeted Brazilian adolescents.^6^ Moreover, this study findings demonstrated that OHRQoL progressively deteriorated as the severity of adolescents’ malocclusion increased. The presence of an ascending gradient in the P-CPQ overall and subscale scores referent to the severity of adolescents’ malocclusion could be explained by the following reasons. First is sample size:^26^ the number of participants based on sample size calculation may impact the distribution of adolescents in each DAI category, thereby influencing the association between severity of adolescents’ malocclusion and P-CPQ scores. The second explanation may be the questionnaire itself: despite being a generic OHRQoL measure, the P-CPQ is a validated tool with reliable psychometric properties tested mainly in pediatric and orthodontic groups.^14^ ^,^ ^19^ A final explanation that could be argued is the fact that cultural and ethnic characteristics,^27^ treatment expectations, and access to orthodontic services impacts the quality of life of young individuals and may also have an impact on the responses provided by their parents/caregivers.^28^

In interpreting the outcome of this study, it is important to bear in mind its limitations. Firstly, the study was conducted with a sample of individuals who were parents/caregivers of adolescents seeking orthodontic treatment at a university clinic. Those individuals were more likely to have higher P-CPQ scores than those who were parents/caregivers of adolescents not seeking treatment, possibly leading to an overestimation of the final results. Secondly, this study presented a cross-sectional design and; therefore, the temporal relationship between the outcome and the main predictor could not be defined. However, adolescents’ malocclusion possibly preceded the outcome avoiding the occurrence of reverse-causality bias.^6^ Finally, although family income has been considered in Poisson regression, this study would have benefited from a multivariate analysis considering parents’ / caregivers’ level of education as a confounding variable.

The results of the present study can serve as a source of information for health planners and governmental authorities in organizing public policies and oral health services.^29^ Patient-reported outcome measures are useful in routine practice to prioritize problems and to identify preferences in health care services.^30^ This information is also relevant for clinicians to inform parents/caregivers about the repercussions of malocclusion on adolescents’ quality of life. Quality of life assessment plays a relevant role in clinical practice as an efficient tool through which health care providers can obtain additional information given by parents / caregivers about the psychosocial impact of oral disorders, such as malocclusion on adolescents’ OHRQoL. Awareness of this information should aid health professionals when referring adolescent patients with the diagnosis of malocclusion to orthodontic treatment.^31^ However, future studies considering different populations with different ethnic and cultural characteristics should be conducted to confirm the findings presented herein. There is also a need for longitudinal studies to furnish more consistent information and assess the long-term effects of adolescents’ malocclusion and orthodontic treatment on the views of their parents/caregivers.

## CONCLUSIONS

Parents/caregivers surveyed in this study reported a negative impact of malocclusion on adolescents’ quality of life. An increased severity of malocclusion is associated with a higher adverse impact on OHRQoL. 
